# Contribution of fracture healing in paediatric DMP zone fracture patients using the pry lever technique

**DOI:** 10.3389/fped.2024.1456136

**Published:** 2024-11-01

**Authors:** Jingyuan Zhang, Hong Cao

**Affiliations:** Department of Traumatic Orthopedics, Renmin Hospital, Hubei University of Medicine, Shiyan, China

**Keywords:** DMP fracture, children, precision shaping, radius, madrone deformity

## Abstract

**Background:**

This study describes the efficacy of applying the prying lever technique to distal radial metaphyseal symphysis diaphysis joining region (DMP) fractures in children, and reports that the technique has an ameliorative effect on the phenomenon of delayed healing and non-union that occurs after surgery for fractures in the DMP region.

**Methods:**

The medical records of 72 children with fractures in the DMP region, treated between December 2017 and December 2023, were retrospectively analyzed. The patients were randomly assigned to either the cut-and-displace group (*n* = 36) or the pry-and-lever group (*n* = 36). Both groups were monitored for time to fracture healing, incidence of delayed or non-union, radiologic outcomes, complications, and functional assessments using the EQ-5D, DASH, and VAS scales.

**Results:**

The follow-up period ranged from 3 to 5 years, with a mean of 26.5 months. After 6 months of follow-up, the incidence of delayed/non-union of fractures was 2.7% and 16.6% in the study and control groups, respectively, and the incidence of delayed healing was significant (*P* < 0.05) in both groups. Additional manipulations or complications caused by delayed healing or non-union were also significantly less in the study group than in the control group. The EQ-5D scale was used to compare the level of surgical satisfaction between the two groups, and the study group had a higher level of satisfaction. Carpal function was significantly improved in both groups compared to the preoperative period, scored using the DASH scale (*P* > 0.05). Postoperative pain level was scored using VAS (*P* < 0.05).

**Conclusion:**

The prying lever technique has the advantages of low impact on periosteal blood supply, simple operation, and fast recovery, which makes it a worthwhile attempt of minimally invasive reduction of fractures in the DMP region in children.

## 1 Introduction

Diaphysis-metaphysis junction zone (DMP) fractures were first proposed by Lieber et al. (1) in 2010 to complement the AO paediatric classification system for long bone fractures (PCCF). It was proposed primarily to provide a higher quality of treatment for DMP zone fractures and to differentiate DMP zone fractures from distal radial metaphyseal fractures. The differences between the two are mainly in the attachment points of the muscles, and the blood supply within the bone. But most importantly there are significant differences in the treatment strategies that need to be adopted between the two (2). In general, the first line of treatment for distal radial metaphyseal fractures in children is closed reduction by manoeuvre, and in principle, without pursuing anatomical reduction and incisional internal fixation, it is possible to rely on the strong bone growth and contouring ability to bring about a satisfactory outcome. For children with distal radial metaphyseal fractures that have failed to be repositioned by manipulation or even with rotations or fractures involving the articular surfaces, a modified Henry approach with palmar internal fixation can be satisfactory.

It is important to note that the distal metaphyseal-diaphyseal (DMP) region lies in the transition zone between the epiphysis and diaphysis. Unlike the epiphysis, which is supplied by a vascular network from the anterior spinoprefectal muscle and the anterior interosseous artery, the DMP region is primarily nourished by smaller blood vessels. Excessive periosteal stripping during tissue separation or secondary nail removal can easily lead to delayed healing or nonunion of fractures in this area. Additionally, the anatomical structure of the radius, with its flat and wide distal end and tubular shaft, poses challenges for stable fixation. The DMP region widens distally and narrows proximally, creating a significant gap between the proximal and distal medullary cavities. This makes it difficult to achieve stable three-point fixation with elastic intramedullary nails, while conservative treatment risks fracture dislocation due to the pulling forces of the brachioradialis, extensor pollicis longus, and associated ligaments.

However, by comparing the results of studies from several tertiary hospitals, we found that some physicians were not able to differentiate between fractures in the DMP region and metaphyseal fractures (3), believing that relatively stable functional reduction and immobilisation could also give satisfactory results for fractures in the DMP region. Most of the published articles also suggest the specificity of the DMP area, but the current treatment mainly consists of external fixation bracing, elastic intramedullary nailing, and plate incision repositioning. Contrary to expectations the efficacy of these methods is only relatively satisfactory and all have some drawbacks (4, 5). In conclusion, due to the specificity of treating fractures in the DMP region and the fact that fractures in children cannot be treated as a reduced version of an adult fracture, most of the current treatment modalities for the DMP region have drawbacks such as the risk of re-displacement and damage to the periosteal blood supply. Therefore, in order to explore an optimal treatment modality applicable to DMP zone fractures, this paper retrospectively analyses the fracture data of 72 cases of difficult-to-replace distal ulnar radius fractures in children in January 2018 2023, and compares the efficacy of prying lever-assisted redundancy and open redundancy of DMP zone fracture under direct vision, which is reported as follows.

## 2 Materials and methods

### 2.1 General information

Inclusion criteria: children diagnosed with a fracture in the DMP region by Lieber's criteria and failed manipulative reduction; children <14 years of age; palmar or dorsal angulation >20° shortening >10 mm; anterior-posterior restriction of rotation ≥30 degrees; and 3 years of postoperative follow-up.

Exclusion criteria: old fractures >2 weeks; open fractures; children with severe vascular and neurological injuries requiring exploration; fractures to the articular surface accompanied by other pathological fractures.

**Figure 1:**
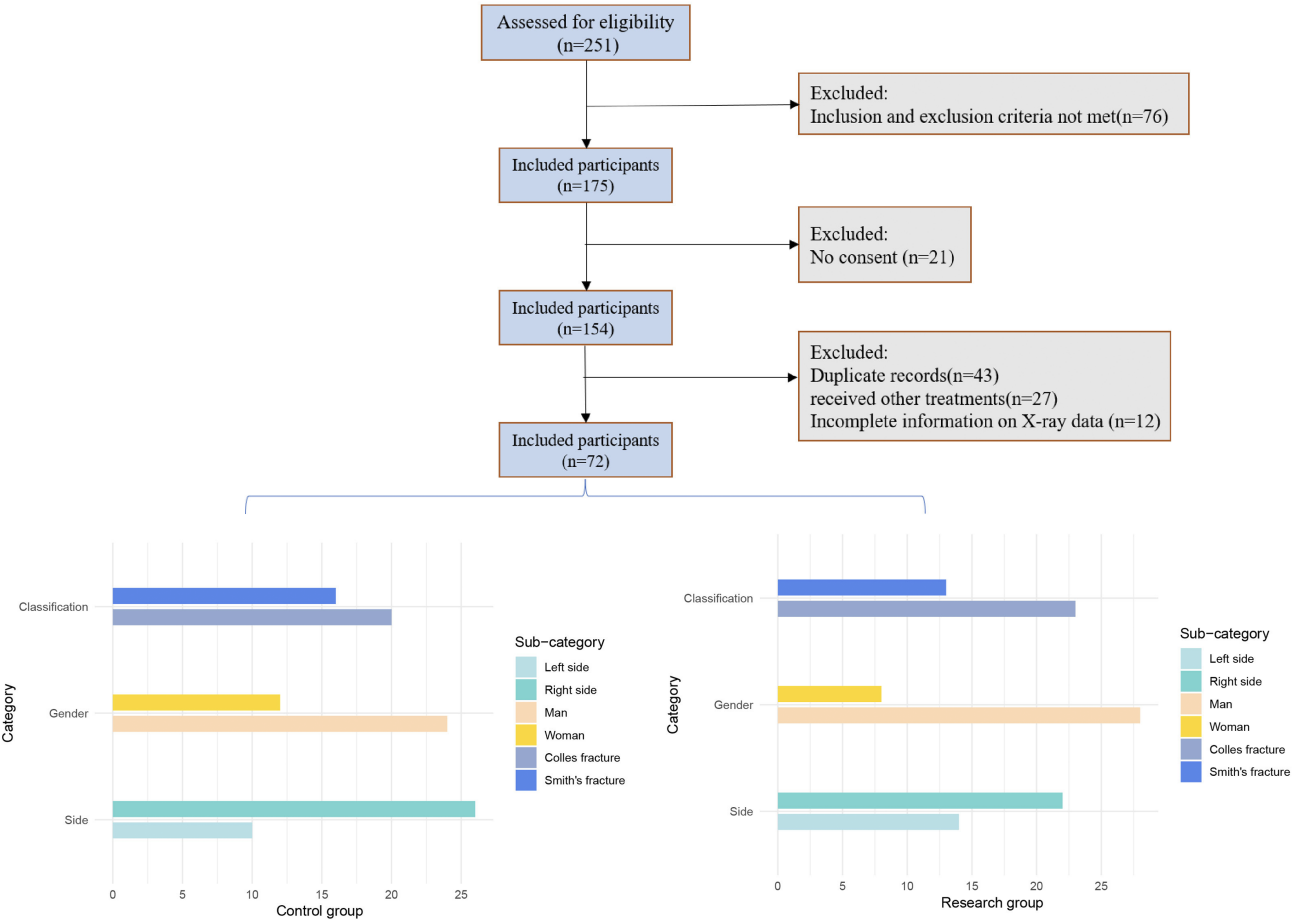
Final population inclusion flowchart.

Initially, 154 patients were included in the study; however, 43 were excluded due to duplicate records (i.e., multiple visits by the same patient). An additional 27 patients were excluded as they received treatments other than open reduction, repositioning, and lever techniques during follow-up. Of the remaining 84 patients, 12 were excluded due to incomplete x-ray data, see Figure 1.

### 2.2 Surgical methods

All surgeries were performed by a single specialist. Initially, all patients underwent manipulative traction, followed by external immobilization using a plaster cast. The criteria for satisfactory repositioning were as follows: (1) The articular surface of the distal radius should align parallel to that of the distal ulna, without any evident misalignment, defects, or discontinuities. The angle of palmar inclination should range between 15° and 20°, and the angle of ulnar deviation should also be between 15° and 20°. (2) The fracture fragments must remain stable without any significant movement or dislocation. (3) The distal radioulnar joint should be symmetrical with the proximal radioulnar joint, displaying no signs of dislocation or subluxation, and the joint space should appear uniform.

In the control group, incision and repositioning were performed under direct vision. The child was placed in a supine position with the forearm abducted on a fluoroscopic rest, and the procedure was carried out under static-aspiration anesthesia. Using the classic Henry approach, the tendon was moved to the radial edge of the incision, and the tendon sheath of the flexor carpi radialis was incised to expose the anterior rotator ani muscle. An L-shaped incision was made along the radial side to strip the anterior muscle, revealing the fracture site. After fracture reduction, stability was checked by rotating the forearm. Once internal fixation was confirmed using C-arm fluoroscopy, the anterior muscle was closed with absorbable suture, unless severely damaged or edematous. If dorsal fracture blocks of the lunate fossa could not be reduced via the palmar approach, a dorsal or combined approach was used. Finally, the wound was closed layer by layer.

**Figure 2:**
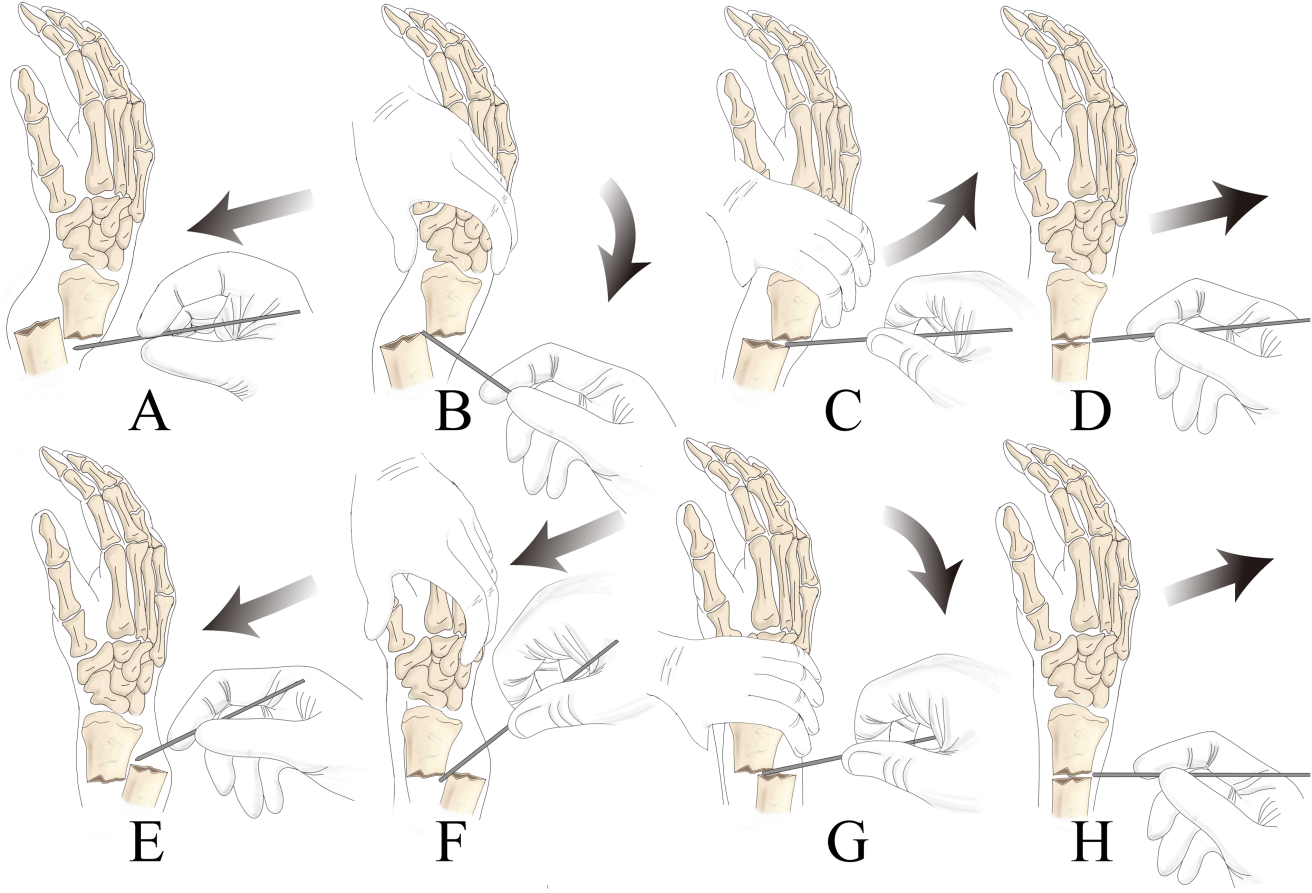
Specific process for leveraging technology **(A)**: percutaneous tip piercing the skin; **(B)** traction or use the blunt end of the Kirschner pin to press the top of the distal bone block to expand the fracture gap, be careful not to use the tip of the forcible entry to damage the soft tissues, to avoid the soft tissues swelling embedded in the fracture site to increase the difficulty of reset; **(C)** extrusion of crushed articular surfaces and the Kirschner pin contact with the distal and proximal fracture, as a prying lever for the reset; **(D)** push the fracture block forward to obtain anatomical reset to ascertain that the fracture position is satisfactory, then the method of pry and pivot reduction of dorsally displaced fractures in the DMP region is similar to the former, see EFGH.

In the study group, a Kirschner-assisted prying lever technique was used for fracture reduction, as outlined in Figure 2. The procedure began with routine disinfection and draping, followed by anesthesia, which was administered either as a brachial plexus block or through a combination of sedation and aspiration. The patient was then positioned under C-arm fluoroscopy to aid in proper alignment and precise guidance during the procedure. For the fracture reduction, a 2.0 mm Kirschner wire was inserted dorsally into the radius at an angle relative to the diaphysis. The specific angle depended on the entry point and the height of the fracture, ensuring optimal placement. Special care was taken to protect the surrounding soft tissues throughout the process. The entry point was cleaned with alcohol gauze or treated with antibiotics, and under fluoroscopic guidance, the needle tip was guided through the skin, while the blunt end was inserted into the fracture gap, minimizing the risk of injury to the dorsal branch of the radial nerve and the cephalic vein.

Using the displaced dorsal fragment as a fulcrum, the assistant applied traction and compression to correct both angulation and rotational deformities of the fracture. To facilitate this correction, traction was applied to the index and middle fingers. The distal end of the Kirschner wire was then maneuvered to gently pry the fracture, correcting posterior rotation and displacement. Given the fragility of pediatric bones, it was essential to control the angle and depth of the wire's penetration to ensure precise alignment with the fracture surface. If lateral displacement occurred simultaneously, an additional Kirschner wire was placed laterally to aid in prying and correcting the alignment in a similar manner.

For fixation, the usual entry points included the attachment of the radial artery at the first extensor tendon compartment and the distal end of the extensor digitorum longus muscle. A transverse Kirschner wire was placed dorsally between the first and second extensor tendon compartments. Two additional Kirschner wires were then inserted from the proximal and distal ends of the fracture at a 20° angle to the axis of the diaphysis. The C-arm fluoroscopy sagittal view was used to confirm that the wires entered the proximal fracture site correctly, ensuring effective fixation.

### 2.3 Clinical evaluation

The study evaluated several clinical outcomes, including operation time, postoperative complications, incidence of delayed/non-union, and the number of additional surgeries required due to delayed/non-union in each group. The RUST score assessed fracture healing, while the VAS scale measured patient pain levels. The DASH score was used to evaluate upper limb disability on the affected side. Patient satisfaction and quality of life were assessed using the EQ-5D-3l questionnaire, a standardized health status tool ranging from 0 (“extremely poor health”) to 100 (“optimal health”).

### 2.4 Statistical methods

The data were analysed using SPSS26.0 statistical software. The normality test was performed by Kolmogorov-Smirnov method. The data conforming to normal distribution were expressed as mean ± standard deviation, and the comparison between 2 groups was performed by independent samples *t*-test, and the comparison between 2 groups was performed by paired *t*-test. Measurement data not conforming to normal distribution were expressed as median (minimum ∼ maximum), and the comparison between 2 groups was performed by Wilcoxon rank-sum test. The Wilcoxon rank sum test was used to compare the two groups. The *χ*^2^ test was used to compare the count data. The difference was considered statistically significant at *P* < 0.05.

## 3 Results

A total of 72 cases were included, age (8.2 ± 5.7) years with a range value of 2–14 years. The cause of injury included high energy injuries (motorbike injuries, smashes), and falls; 24 on the left side and 48 on the right side. All were new closed fractures. x-rays suggested that the patients had unstable fractures involving the growth plate and should not be over-manipulated. The time from injury to surgery was (2.3 ± 1.1) hours, with a range value of 1 to 4 h. Fractures were predominantly repositioned using manipulation before redisplacement, with poor anatomical relationship-s. Comparison of the general data of the 2 groups is shown in Table 1, and is comparable.

**Table 1:** Comparison of general information between the two groups (*n* = 72).

		Research group	Control group	*t* (*Z*, *χ*^2^)	*P*
Age		8.8 ± 6.2	7.5 ± 5.6	0.934	0.354
Side	Left side	14	10		
	Right side	22	26	1.000	0.317
Gender	Man	28	24		
	Woman	8	12	1.108	0.293
Classification	Colles fracture	23	20		
	Smith's fracture.	13	16	0.520	0.471
Time from injury to surgery		2.3 (1–3)	2.5 (2–4)	0.044	0.965

K-wire-assisted reduction was successfully performed in all 36 cases, with a follow-up period ranging from 3 to 5 years (mean: 26.5 months). None of the patients developed complications such as needle track infection or compartment syndrome postoperatively. A *χ*^2^ test comparing postoperative complications (e.g., needle track infection, tenosynovitis, and flexor tendon injury) between the study and control groups showed no significant difference (*P* > 0.05). Two patients in the control group experienced suspected flexor tendon irritation due to needle puncture at the watershed line, leading to tenosynovitis and flexor tendon injury. These conditions improved after weight-bearing avoidance, pressure relief, and rehabilitation exercises. In the study group, two patients experienced radial nerve branch damage due to K-wire fixation, resulting in numbness in the crotch area. Following 3 weeks to 3 months of nerve rehabilitation, most patients regained function. The outcomes are shown in Tables 2, 3.

**Table 2:** Postoperative complications and additional operations.

		Research group	Control group	χ^2^	*P*
Complication	Radial nerve injury	2	0	5.306	0.139
	Tendon rupture/irritation	0	2		
	Delayed healing	1	3		
	nonhealing	0	3		
Additional operations due to delay/non-healing	Autologous bone graft	0	2	2.585	0.782
	internal fixation by incision	1	3		
	Bone stimulator	1	1		
Incidence of delayed/non-healing (cases/%)		1 (2.7)	6 (16.6)	3.956	0.047

**Table 3:** Comparison of DASH, VAS, and CWS scores between two groups of patients (*n* = 72, x ± s points).

		Research group	Control group	*t*	*P*
DASH	Pre-treatment	40.8 ± 4.3	41.7 ± 5.6	0.765	0.447
	Post-treatment	9.0 ± 0.9	9.5 ± 1.4	1.802	0.076
VAS	Pre-treatment	6.5 ± 2.6	7.1 ± 3.4	0.627	0.534
	Post-treatment	3.1 ± 1.1	4.2 ± 0.8	4.852	0.001
EQ-5D	Pre-treatment	46.4 ± 4.7	44.9 ± 8.4	0.935	0.353
	Post-treatment	94.3 ± 4.7	89.7 ± 3.1	4.902	0.001

Fracture healing time was compared using an independent sample *t*-test, showing no significant difference between the study group (7.8 ± 2.1 weeks) and the control group (*P* > 0.05). One patient in the study group had delayed healing, with improvement seen after more than 6 months (*P* > 0.05). Upper limb disability and wrist joint function were assessed using the DASH scale, and the independent sample *t*-test showed no significant difference between the groups (*P* > 0.05). Patient satisfaction, evaluated using the EQ-5D scale, also showed no significant difference between the groups (*P* > 0.05). Pain levels were assessed using the VAS, and paired *t*-tests showed a significant improvement in postoperative scores compared to preoperative scores (*P* < 0.05), as shown in Table 3.

**Figure 3:**
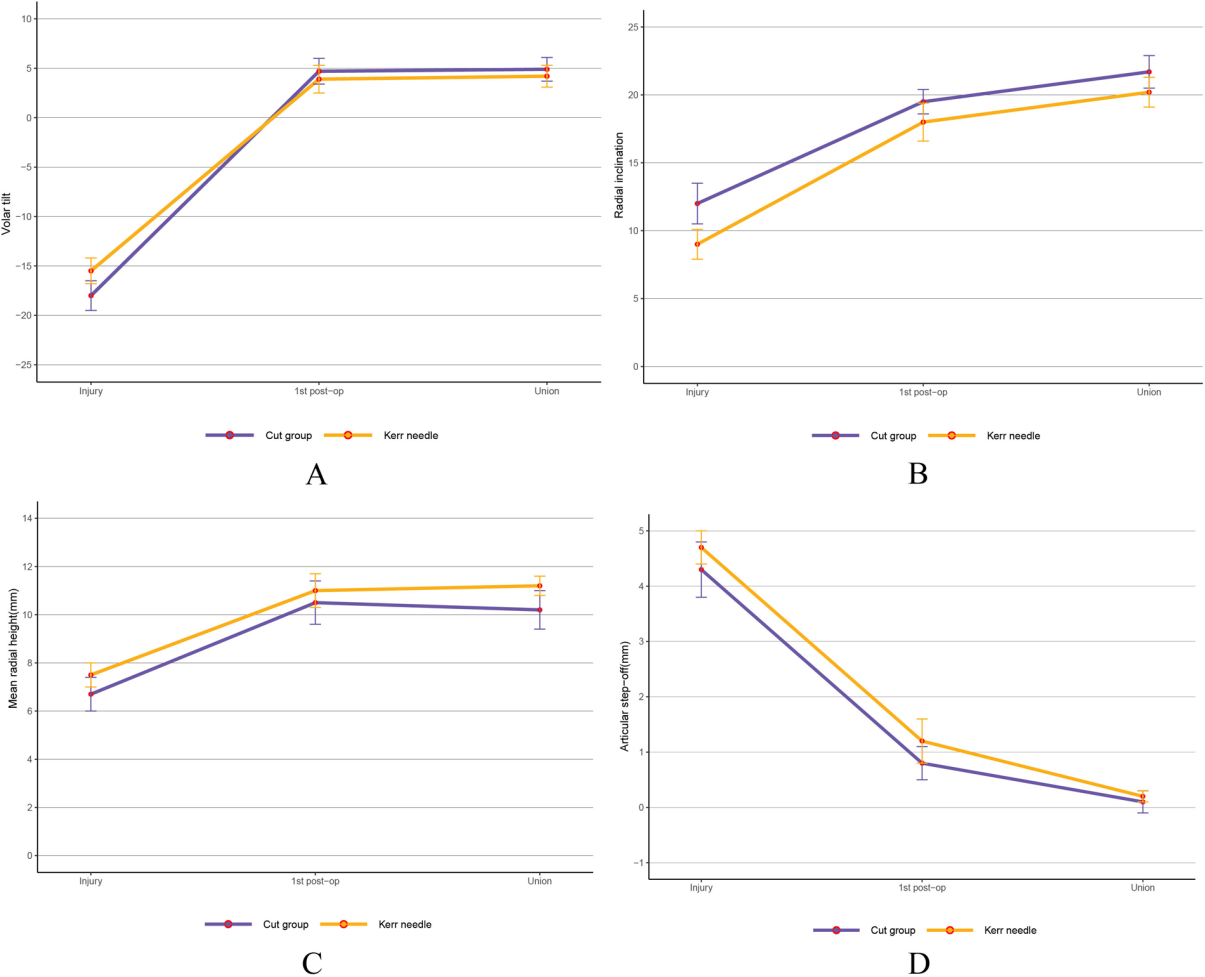
Preoperative, postoperative, and radiological results after complete fracture healing. **(A)** Volar tilt **(B)** Radial inclination. **(C)** Mean radial height. **(D)** Articular step-off. There was no statistically significant difference between the two groups.

For RUST scores, repeated measures ANOVA showed significantly higher scores at 3 months post-surgery and at final follow-up compared to preoperative scores (*P* < 0.05), with the study group scoring higher than the control group (*P* < 0.05) (Table 4). The K-wire incision was approximately 3 mm, smaller than in the control group, with no needle track infections. This may be attributed to the use of alcohol gauze for local skin disinfection and the application of antibiotics to the needle during insertion. Radiological parameters (radial height, radial inclination, and palmar tilt) were assessed at injury, the first postoperative follow-up, and at fracture healing (Figure 3), with no significant differences between the two groups (*P* > 0.05). Independent sample *t*-tests were used for group comparisons at each time point.

**Table 4:** Comparison of healing related indicators.

		Research group	Control group	*t*, *Z*	*P*
Total length of incision (mm)		2.3 ± 0.9	65 ± 12.3	22.736	0.001
Fracture healing time (months)		2.3 ± 0.8	5.7 ± 2.3	8.377	0.001
Delayed healing time (>6 months)		0.8 ± 0.4	2.1 ± 0.8	8.721	0.001
RUST score	Preoperative	0.8 ± 0.7^a^	0.9 ± 0.6	0.651	0.517
	3 months after surgery	2.8 ± 0.9	2.1 ± 0.7	3.684	0.001
	Last follow-up	3.3 ± 1.2^a^^,b^	2.5 ± 1.1^b^	2.949	0.001
	t, *P*	t^a^ = 10.79 *P*^a^ = 0.001	t^b^ = 7.66 *P*^b^ = 0. 001		

Note: Compared with preoperative fracture healing.

^a^ *P* < 0.05; compared with fracture healing over the same period.

^b^ *P* < 0.05.

## 4 Discussion

**Figure 4:**
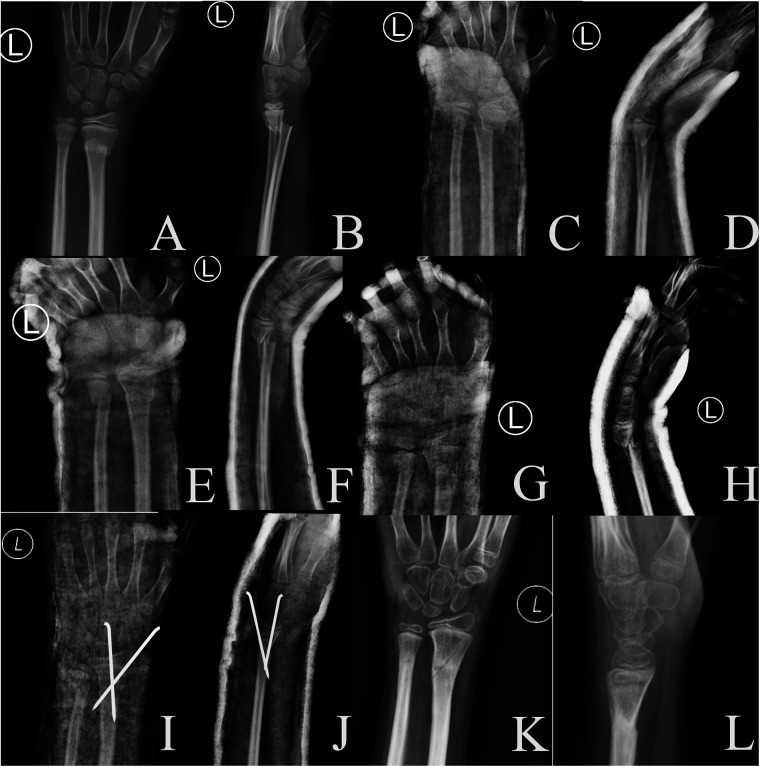
X-rays of an 11-year-old male boy who suffered a fracture of the distal end of the left radius and ulna due to a fall. **(A,B)** preoperative anteroposterior and lateral radiographs; **(C,D)** first reduction anteroposterior and lateral radiographs; **(E,F)** second reduction anteroposterior and lateral radiographs; (**G,H**) third reduction anteroposterior and lateral radiographs; **(I,J)** post-operative reduction with prying lever technique Anteroposterior and lateral radiographs; **(K,L)** Anteroposterior and lateral radiographs after removal of K-wires after healing.

**Figure 5:**
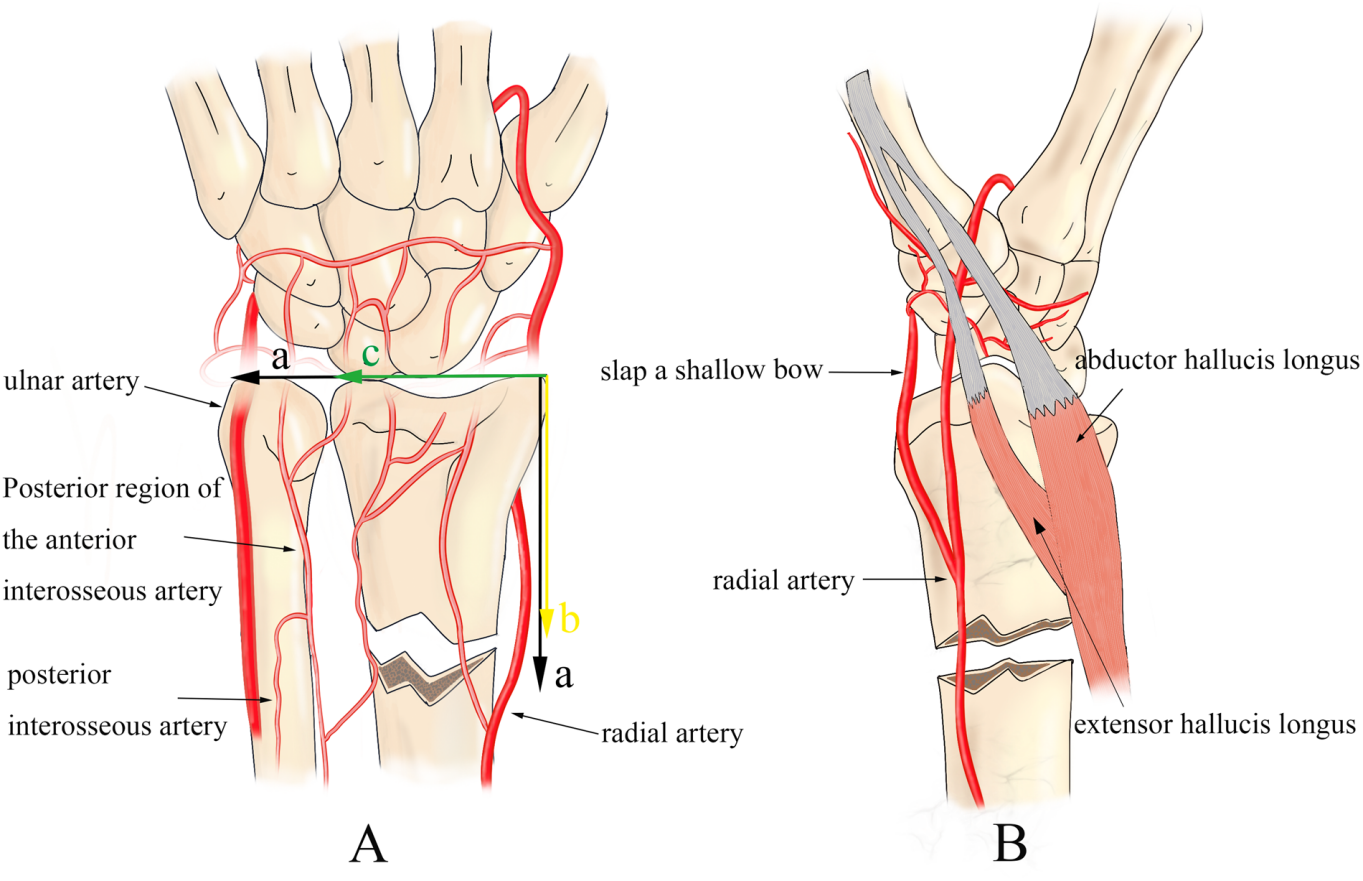
Definition and characteristics of DMP zone fracture: **(A)** take the sum of the radial ulnar epiphyseal plate as **(a)**; the distance of the epiphyseal plate from the fracture line as **(b)**; the width of the radial epiphyseal plate as **(c)**. DMP fracture is considered when a > b > c (7). (**A,B**) The blood supply of the distal radius mainly includes the superficial palmar arch, ulnar and radial arteries, and the anterior and posterior interosseous arteries, and it can be concluded that the reticular blood supply of the metaphysis is significantly richer than that of the diaphysis. Moreover, the fracture in the DMP region is susceptible to the pulling of the bunion and extensor digitorum longus muscles, thus leading to fracture redisplacement and delayed, non-union.

When the blood is not circulating properly, stasis occurs and cannot be resolved; if the stasis is not resolved, the bone cannot heal. Therefore, attention to blood supply should be carried out throughout the treatment of fractures in the DMP region. Fractures in the DMP region occur closer to the diaphysis, so they are characterised by a smaller contact area between the broken ends, poor local blood supply, difficult to reset, and a low maintenance rate compared with distal metaphyseal fractures (6), see Figures 4, 5. Delayed healing should be considered when the fracture in the DMP region does not show signs of healing within 6 months and the fracture gap is not reduced on x-ray; non-union should be diagnosed when there is no healing trend after 6 months or when x-ray shows obvious bone loss or pseudoarthrosis formation (8).

By comparing the treatment modalities of more than 10 tertiary hospitals (9), it was found that the treatment of fractures in the DMP region is not paid much attention to at present, and there are even competent doctors who only use similar treatment modalities and procedures as those for metaphyseal fractures to diagnose and treat them. For distal radial metaphyseal fractures, most of the patients only need 3–5 weeks of plaster immobilisation after manipulative reduction to achieve the expected results (10, 11). However, the DMP region is in the transition zone between the flattened broad bone and the tubular bone, and the peripheral arterial network is far less rich in nourishment than that of the metaphysis. Moreover, the characteristics of the radius bone are far wide and narrow, which will lead to a shorter diameter and smaller area of contact between the broken ends of the DMP area, so it can not be treated as a fracture of the distal metaphysis of the radius bone. Some scholars have proposed that patients with non-displaced fractures in the DMP region can be directly fixed externally and choose splints or long-tube casts according to the patient's needs, in which children with an angle of <10° in any direction and younger than 15 years of age have a higher probability of obtaining satisfactory contouring results (12). This indirectly indicates that the indications for conservative treatment in patients with fractures in the DMP region are strict and only applicable to patients with lesser degree of displacement and should not be confused with distal radial metaphyseal fractures, which may cause complications such as nonunion, delayed healing, and nonunion.

**Figure 6:**
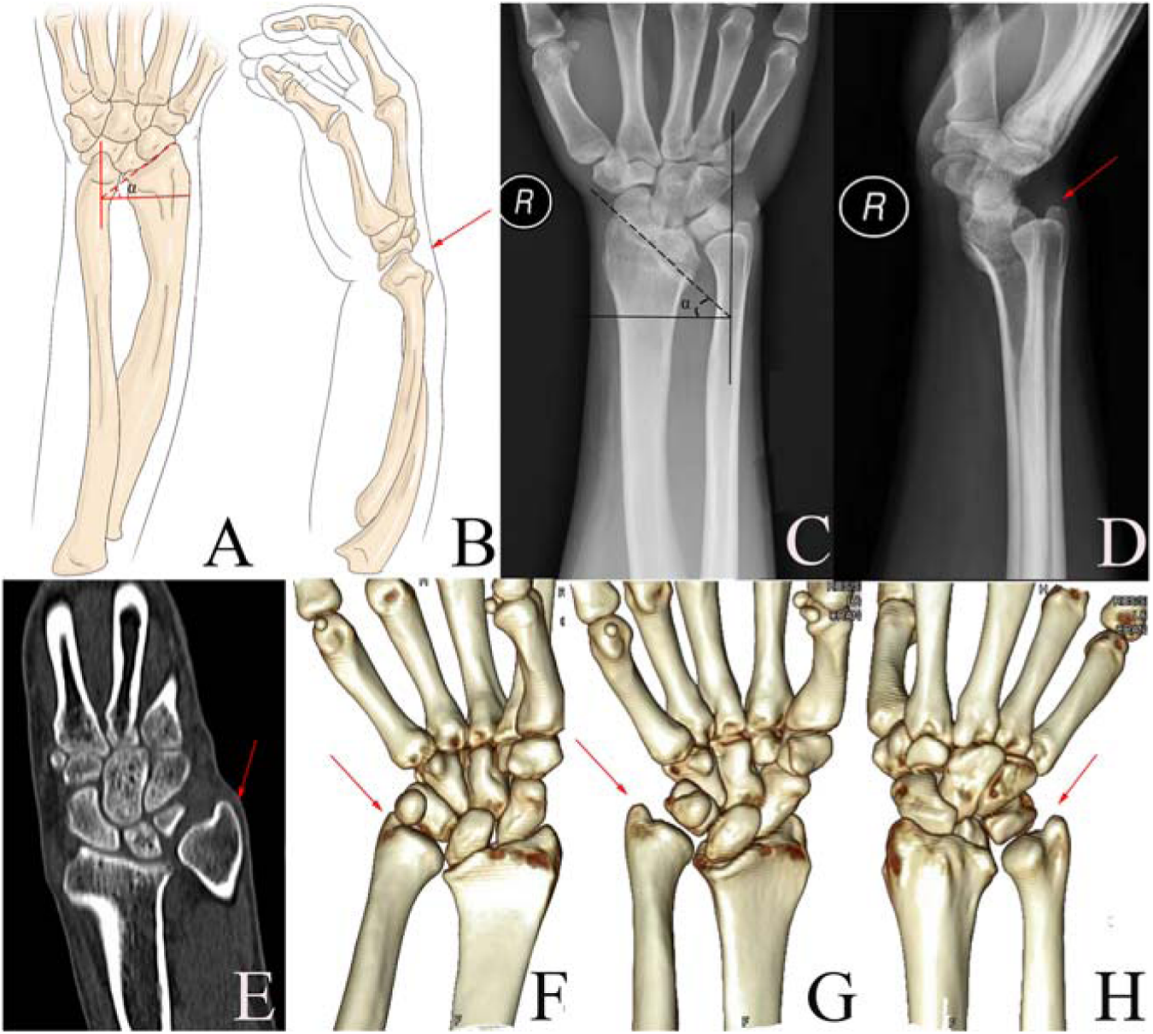
Child, female, 19 years old, with a habitual dislocation of the ulna as a result of epiphyseal damage caused by multiple failed fracture repositionings in early childhood, leading to the formation of a madelung deformity in later life. **(A,C)** Lunate fossa angle α greater than or equal to 35° is the more sensitive indication of Madelung's deformity, the forearm is short and flexed, the wrist is weak, the radius is too short compared to the ulna, the distal ulna is subluxated dorsally, and the subluxation is easy to be reset but cannot be sustained; **(B,F)**: the radius is shorter compared to the ulna; **(D)** the distal end of the radius articulation is offset to the palmar side, and the ulna is offset dorsally, and there is a defect in the angle of palmar inclination on the ulnar side; **(E,G,H)** a combination of delayed medial palmar development of the radius with a semi-dislocation of the ulna can be seen.

In this study, although there was no significant difference between the 2 groups in terms of functional recovery of the wrist, the pry lever-assisted technique was more advantageous in terms of healing time, number of cases of delayed healing, and the incidence of re-displacement compared with direct view incisional repositioning. The use of this technique has little or no effect on the blood supply to the DMP area and significantly improves the incidence of fracture non-union. During follow-up, two patients developed nerve damage, and one experienced the rare complication of Madelung's deformity (Figure 6). Radial nerve injury is typically caused by temporary factors such as pulling, compression, or disuse, and often resolves spontaneously in the early stages. Song et al. advise against early nerve decompression, as neurological symptoms may result from local edema or hematoma compression. In this study, two patients with radial nerve injuries exhibited persistent neurological symptoms with no significant improvement after 2–3 weeks of observation. Although there was some improvement after nerve release and decompression, residual symptoms persisted during follow-up. Based on these findings, we recommend immediate surgical exploration for patients who continue to experience neurological symptoms 24–36 h postoperatively and whose symptoms are not effectively controlled by active treatment.

Madelung deformity is often characterised by palmar flexion and ulnar deviation of the wrist, shortening of the radius, abnormal alignment of the carpal bones, and an abnormal distribution of joint forces (13, 14). The key to preventing and treating Madelung deformity is early intervention or avoidance of re-displacement after the first repositioning, therefore less invasive therapies are advocated. There are no relevant reports on the reduction of the incidence of Madelung deformity by percutaneous Kirschner's needle prying and dialling repositioning. However, a large body of evidence emphasises that acceptable functional repositioning with satisfactory periosteal blood supply can avoid the occurrence of Madelung deformity (15). The prying technique is less invasive and interferes less with the periosteal blood supply, so early prying intervention can effectively reduce its incidence (16).

In our group, 36 cases were treated with incisional reduction, and all of them were worse than prying-assisted reduction in terms of fracture healing. In addition, it has been reported that conventional elastic intramedullary nailing in the DMP region also fails to bring satisfactory results (17, 18). Firstly, the attachment of the brachioradialis, extensor digitorum longus and extensor hallucis longus muscles as well as ligamentous pulling in the vicinity of this region make it difficult to access the proximal medullary cavity through a retrograde approach. This poses a problem for subsequent intramedullary fixation using pre-curved elasticated nails crossed in the medullary cavity. Secondly, even if the fixation is satisfactory, the elasticity of the elastic nail itself will result in a high risk of re-dislocation due to the short distal force arm, which is unable to counteract muscular pulling and form an effective “three-point support”. A solution to this problem has been proposed by placing a de-elasticated pre-curved elastic nail in a parallel path with the backbone (19). Lu Jinyuan et al. treated 25 children with parallel elastic intramedullary nailing (20), which confirmed the feasibility of internal fixation of fractures in the DMP region by opening the proximal radius 2–4 cm from the articular surface, suggesting that the use of modified intramedullary nailing techniques can be used to treat fractures in the DMP region. However, these techniques rely on proficiency and manipulation skills, and if not performed properly de-elasticising the nail may lead to stress concentration at the bend of the intramedullary nail, increasing the risk of nail stem fracture; and the entry and secondary removal of the nail may strip more soft tissue, leading to delayed healing.

It has also been recently demonstrated that with the widespread use of elastic nailing, the probability of experiencing delayed fracture healing and non-union has increased from 0.3%–3% to more than 7% (21). In addition to this, the window of the safety zone of the paracentesis approach is only 2–3 cm away from the posterior interosseous nerve, which is relatively poor in terms of extrapolation, although the operation of Lu Jingyuan et al. has a lesser chance of nerve injury. In this study, the control group's incisional repositioning was able to separate the tissues under direct vision, which was satisfactory in restoring the dorsal inclination and avoiding nerve injury in the postoperative period. However, its biggest problems were the need to strip a large amount of periosteum and the controversial repair of intraoperative anterior rotator ani muscle injury.

Goorens CK et al. believed that repairing the anterior rotator ani muscle was only advantageous in early pain management (22); Shi Fenglei et al. believed that it might have no effect on the recovery of late wrist function regardless of whether it was repaired or not (23); and other scholars (24) believed that it needed to be repaired for its greater impact on the flexor tendons and the function of the wrist joint rotation anteriorly.

Therefore, it can be concluded that there is no optimal fixation treatment for fractures in the DMP region, and most of the commercially available options are not effective in resolving non-union. Although percutaneous Kirschner's pin pry-pull reduction has been applied to metaphyseal fractures many years ago, and the efficacy is good. However, fewer studies have been conducted to observe the efficacy in fracture healing in the DMP region. In our study, the prying-assisted technique was gentler than incision, which significantly improved periosteal blood supply, reduced the number of fracture redisplacements and unnecessary additional manipulation, in addition to effectively reducing pain and the rate of secondary incision, and seemed to be more advantageous in restoring radial length. Therefore, the satisfactory results of early application of pry-pull reduction in DMP zone fractures indirectly suggest that it may become the best and preferred treatment option for DMP zone fractures.

The advantages of this operation are: (1). Correction of deformity is fast and effective, which can reduce the braking time of the affected limb, and can be satisfactory in the prevention of long-term complications (9, 12) and reduction of fluoroscopic radiation. (2). Precise action on the fracture, so that the absorption of traction force by the surrounding muscles and soft tissues is minimised, avoiding soft tissue swelling and the possibility of soft tissue embedded in the fracture end during the reset of the fracture in older children (12, 25). (3). In line with the concept of minimally invasive, the actual reduction operation has an incision of only 2–3 mm. The small incision and satisfactory alignment are more likely to gain the trust of the family and enhance the image of the hospital. (4). Low cost and easy to start. The price of Kirschner's needle is lower than that of intramedullary nails, plate placement and external fixators, even if the cost of local anaesthesia is added to the outpatient clinic to take out the cost of the patient's burden is not heavy. (5), the operation is more gentle, minimise the damage to the periosteum blood supply, so that the blood supply of the fracture end is more abundant, which is conducive to fracture healing.

The details of this operation are: (1), when the pry bar into the fracture gap, should be in the fracture broken end of the sliding touch to the tip of the needle touching the peripheral wall of the medullary cavity appeared obvious resistance and feel the obvious sense of obstruction to determine the pry point. (2), According to the thickness of the bones shown in the x-ray and the age of the child, choose a pry bar with a diameter of 1.5 to 2.0 mm. Because the diameter less than or equal to 2.0 mm smooth Kirschner pin even if inadvertently through the epiphysis, epiphyseal damage is not serious, but too small anti-stress is too poor to increase the difficulty of prying; (3), Continuous traction should be applied before the end of fixation to avoid positional loss. (4), 6-pin biplane reduction method can be used to fix the comminuted fracture, the first pin from the radial tuberosity into the radial long axis at an angle of 40–60°, and dorsal 10°, until the proximal medial cortex of the radius; the second pin from the proximal radial fracture line into the radial, fixed to the radius ulnar bone block, to avoid loss of the position of the bone; the third pin from the head of the ulnar head through the radial articular endosteal bone fragments, the radial tuberosity out. The other 3 pins are reinforced in the direction of the original 3 pins, constituting a bi-planar three-dimensional stabilisation structure, which can maintain the height of the radius and prevent the position of the bone fragments from changing. (5), Through the radial styloid, more than 2 needles should be used and cross-fixed in a fan shape to improve the strength and the needles should be inserted as close as possible to the end of the radial styloid in order to prevent injury to the superficial branch of the radial nerve.

This study has limitations: This study primarily reviewed and analyzed previously collected patient data, with a relatively small sample size. The study design and statistical methods were focused on detecting more pronounced effects, which may have limited the statistical power to identify smaller differences. Due to the sample size constraints, the study did not stratify patients by age, preventing the exploration of potential differences in fracture healing or surgical outcomes across age groups. Additionally, we did not differentiate between fully displaced and partially displaced fracture types. Future research will aim to include a broader range of control groups, such as minimally invasive techniques, to more comprehensively compare the outcomes of different treatment options.

In conclusion, delayed fracture healing and non-union are common and serious complications of radial DMP zone fractures. Smooth blood circulation is the prerequisite for smooth meridians, strong muscles and bones, and flexible joints. Therefore, when there is a tendency of delayed healing or non-union of radial fracture in clinic, it should be considered whether the operation method is inappropriate or not. Is it possible that the treatment of DMP fractures with the same procedure as metaphyseal fractures is interfering with the periosteal blood supply and affecting the haematoma mechanism?This returned to the starting point of this study, and we found that minimally invasive pry-pull reduction of DMP zone fractures can restore fracture stability without disturbing the blood supply and with less surrounding tissue damage and bone destruction. This technique is simple and easy to perform and can be used as an extrapolatable method to reduce postoperative delayed healing and nonunion of fractures in the DMP region.

## Data Availability

The original contributions presented in the study are included in the article/Supplementary Material, further inquiries can be directed to the corresponding author.
